# Supplementary material for: ATML1-GIR1-TPL/TPR transcriptional repression module controls glucosinolates and giant cells in Arabidopsis thaliana sepals
Source: bioRxiv. 2026 Jun 6:2026.06.03.724713. Preprint. [Version 1] doi: 10.64898/2026.06.03.724713 (PMC13252061; doi:10.64898/2026.06.03.724713)
Supplement: Supplement 1 — Fig. S1. The gir1 and gir1;gir2 mutants exhibit similarly enhanced giant cell formation. Fig. S2. Overexpression of GIR1 results in rescue of giant cell phenotype of gir1 mutants. Fig. S3. ATML1 STAD functions as a transactivation domain. Fig. S4. Purification of recombinantly expressed GIR1 and ATML1 STAD wild-type and mutant proteins. Fig. S5. Western blot of GIR1 proteins expressed in Y2H assays. Fig. S6. Heatmap from RNA-seq data showing three biological replicates from each genotype. Fig. S7. RNA-seq data for additional aliphatic GSL biosynthesis genes. Fig. S8. RNA-seq data for additional indolic GSL biosynthesis genes and other GSL pathway genes. Fig. S9. MALDI-MS imaging of the sepal epidermis indicates high 4MSOB GSL levels in gir1 mutants. Table S1. Steady state constants and binding affinity of GIR1 to ATML1 STAD and atml1 STADD589L. Table S2. Summary of expression of GSL biosynthesis genes in gir1 sepals. Table S3. Names and chemical properties of GSLs described in this study. Table S4. Oligonucleotides used in this study. [file media-1.pdf]

Supplemental Information for:

ATML1-GIR1-TPL/TPR transcriptional repression module controls glucosinolates and giant cells in *Arabidopsis thaliana* sepals

Lauren E. Apprill[a,b], Bilal Ahmad[b], Aytug Ulutas[b,c], Amanda Agosto Ramos[d], Sumin Na[e], Samira R. Laytimi[b], Ayianna K. Bailey[b], Adara L. Warner[b], Titus R. Neumann[b], Young-Jin Lee[e], Brandon L. Garcia[a], Daniel J. Kliebenstein[d], and Kathrin Schrick[a,b,c,*]

[a]Department of Biochemistry and Molecular Biophysics, Kansas State University, Manhattan, KS 66506, USA

[b]Division of Biology, Kansas State University, Manhattan, KS 66506, USA

[c]Interdepartmental Genetics Program, Kansas State University, Manhattan, KS 66506, USA

[d]Department of Plant Sciences, University of California, Davis, Davis, CA 95616, USA

[e]Department of Chemistry, Iowa State University, Ames, IA 50011, USA

*Kathrin Schrick

Email: kschrick@ksu.edu

This PDF file includes:

SI Methods

Figures S1 to S9

Tables S1 to S4

Legends for Datasets S1 to S9

SI References

Other supporting materials for this manuscript include the following:

Datasets S1 to S9

SI Methods

Mass Spectrometry Imaging

Sepals were dissected under a stereo microscope, and the abaxial side of the sepal was mounted facing up on a glass slide using a double-sided conductive carbon tape. Three sepals from each genotype were placed on the same slide to minimize batch effects. 9-aminoacridine (9-AA; 15 mg/mL in MeOH: H_2O (30:70, v/v) was applied as a matrix using a TM sprayer (HTX Technologies, Chapel Hill, NC), and Au was sputter-coated for 20 s at 40 mA (Cressington 108; Ted Pella, Redding, CA) to provide surface conductivity. Matrix-assisted laser desorption/ionization mass-spectrometry imaging (MALDI-MSI) was performed using a QExactive HF Orbitrap MS (Thermo Scientific, San Jose, CA) with a MALDI source (Spectroglyph, Kennewick, WA) equipped with a 349-nm laser (Explorer One; Spectra Physics, Milpitas, CA). Data were collected in negative-ion mode for the m/z range of 400–500 with a mass resolution of 120,000 at m/z 200 to target the GSLs signals. Raster steps of 20 µm were used, considering the small size of sepals. Raw data were converted to imzML files using ImageInsight (Spectroglyph), and imzML files are loaded into the MSiReader (North Carolina State University; Raleigh, NC) software [1] to generate MS images, and METASPACE [2] was used for annotation with a false discovery rate (FDR) threshold of 10% and a mass tolerance of ± 3 ppm.

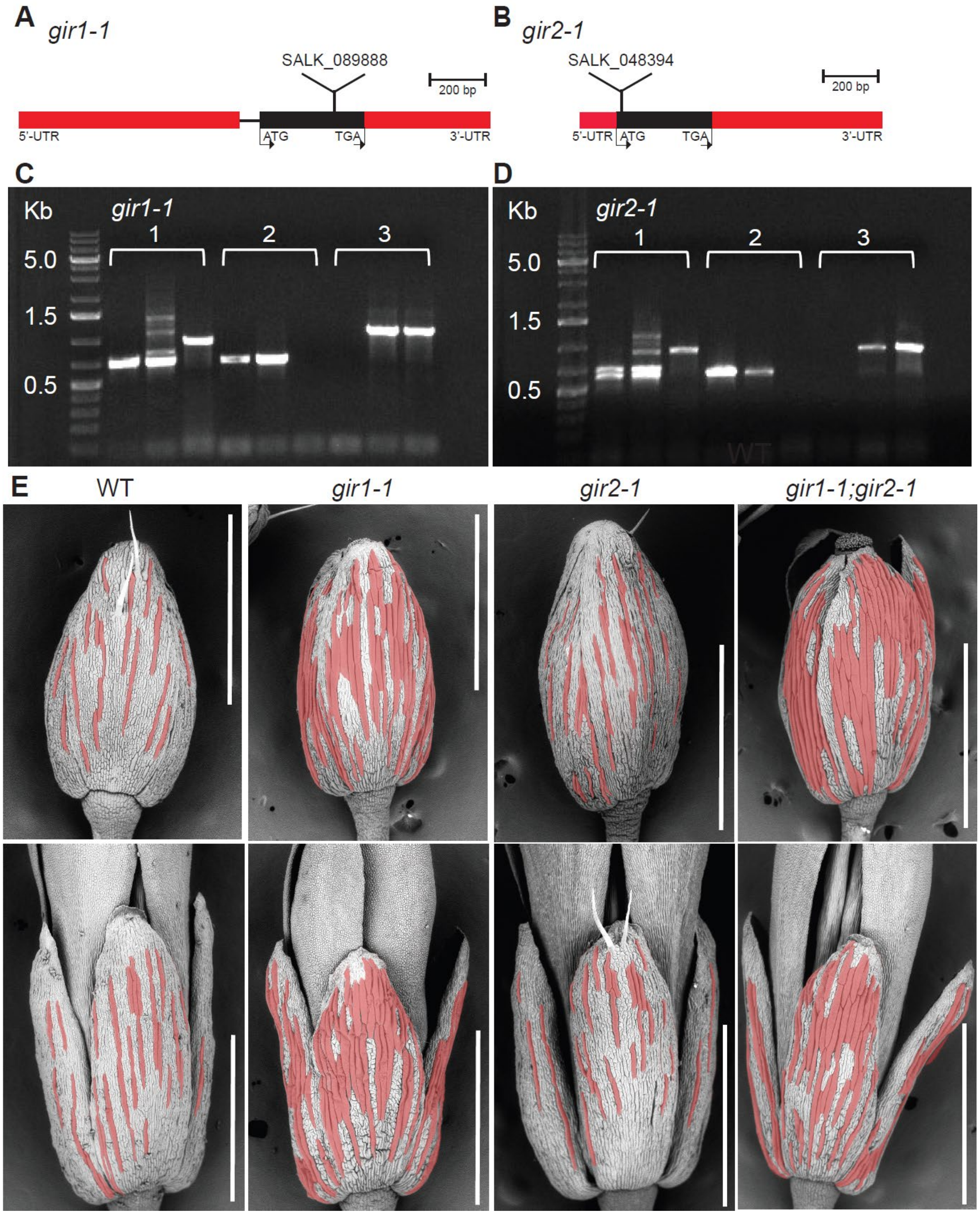

Fig. S1. The *gir1* and *gir1;gir2* mutants exhibit similarly enhanced giant cell formation.

(A) and **(B)** Schematic diagrams illustrating genomic regions encoding GIR1/At5g06270 (left) and GIR2/At3g11600 (right). The positions of the respective T-DNA insertions are indicated for *gir1-1* and *gir2-1* alleles. Untranslated regions (UTRs) are depicted in red, exons and introns are shown by black thick and thin lines, respectively.

(C) and **(D)** Genotyping of *gir1-1* (SALK_089888) and *gir2-1* (SALK_048394) T-DNA insertion mutants given in **(A)** and **(B)**. **Panel 1**: Amplicons from three-primer PCR with corresponding gene-specific (RP and LP) and left-border (LB) primers; **Panel 2**: Amplicons from two-primer PCR with LP and the RP gene specific primers; **Panel 3**: Amplicons from the two-primer PCR with the gene-specific primers. In each panel from left to right are shown amplicons from the mutant, heterozygote, and wild-type DNA. Oligonucleotide primers are given in **Table S4**.

(E) Giant cell phenotypes on sepals from wild type (WT) in comparison to *gir1, gir2,* and *gir1;gir2* mutants. Giant cell formation is similarly enhanced in *gir1 and gir1;gir2* mutants, while *gir2* mutants display a wild-type phenotype. Scanning electron microscopy (SEM) images of stage 12 (top panel) and stage 14 (bottom panel) flowers. Giant cells are falsely colored red. Bar = 1 mm. Quantification of giant cell numbers per sepal and giant cell width is given in **Fig. 1C**.

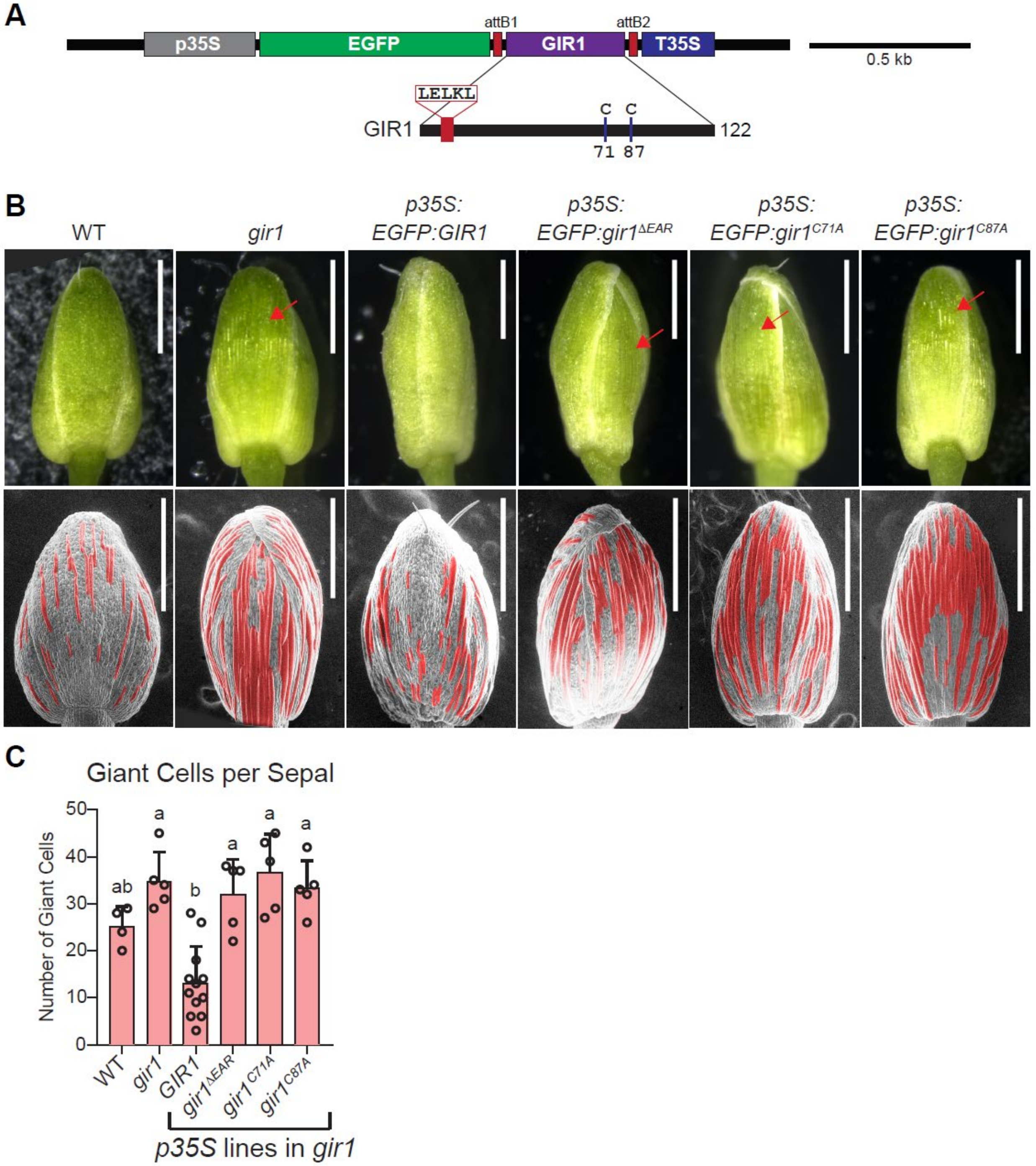

Fig. S2. Overexpression of *GIR1* results in rescue of giant cell phenotype of *gir1* mutants.

(A) Schematic of the transgenic CaMV 35S promoter (*p35S*) constructs for *GIR1* wild-type and mutants affecting the function of the EAR motif (*gir1*$^{\Delta EAR}$) and predicted Zn finger (*gir1*^{C71A} and *gir1*^{C87A}). **(B)** Stereo microscopy images of flower buds (stage 12) (top) and matching scanning electron microscopy (SEM) images (bottom) with constructs indicated in **(A)**, in comparison to wild type and *gir1* controls. Giant cells

are false-colored red. The wild-type *GIR1* construct rescued *gir1* phenotype while *gir1* mutants (*gir1*$^{\Delta EAR}$, *gir1*^{C71A}, *gir1*^{C87A}) failed to rescue. Bar = 500 μm. **(C)** Number of giant cells per sepal were quantified for the genotypes shown in **(B)**. SD are indicated for n=4-12 biological replicates. Significant differences were assessed by one-way ANOVA, Tukey's test, and are indicated by letters (p value < 0.05).

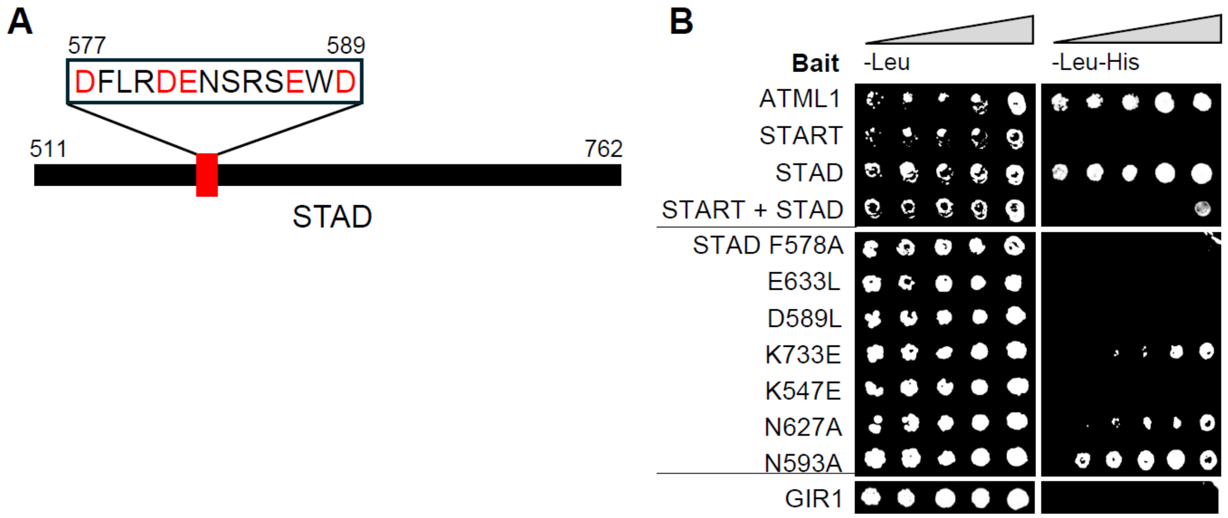

Fig. S3. ATML1 STAD functions as a transactivation domain.

(A) Schematic of ATML1 STAD is illustrated, highlighting a 13 amino acid acidic patch. Acidic amino acids, Asp (D) and Glu (E), are indicated in red. **(B)** Yeast two-hybrid (Y2H) bait autoactivation assay reveals that ATML1 STAD is an auto-activator and that specific residues in the STAD acidic patch (F578, D589) and other regions of STAD are critical for this function. Haploid yeast expressing bait plasmids with full-length ATML1, START, STAD, START+STAD, STAD mutants, or GIR1 alone were tested for transcriptional activation without prey plasmids. Growth on permissive media (-Leu) indicates presence of the bait plasmid. Growth on selective media (-Leu -His) signifies autoactivation, or transcriptional activation without requiring the prey plasmid (which contains an activation domain). Four-fold dilutions of yeast are indicated right to left.

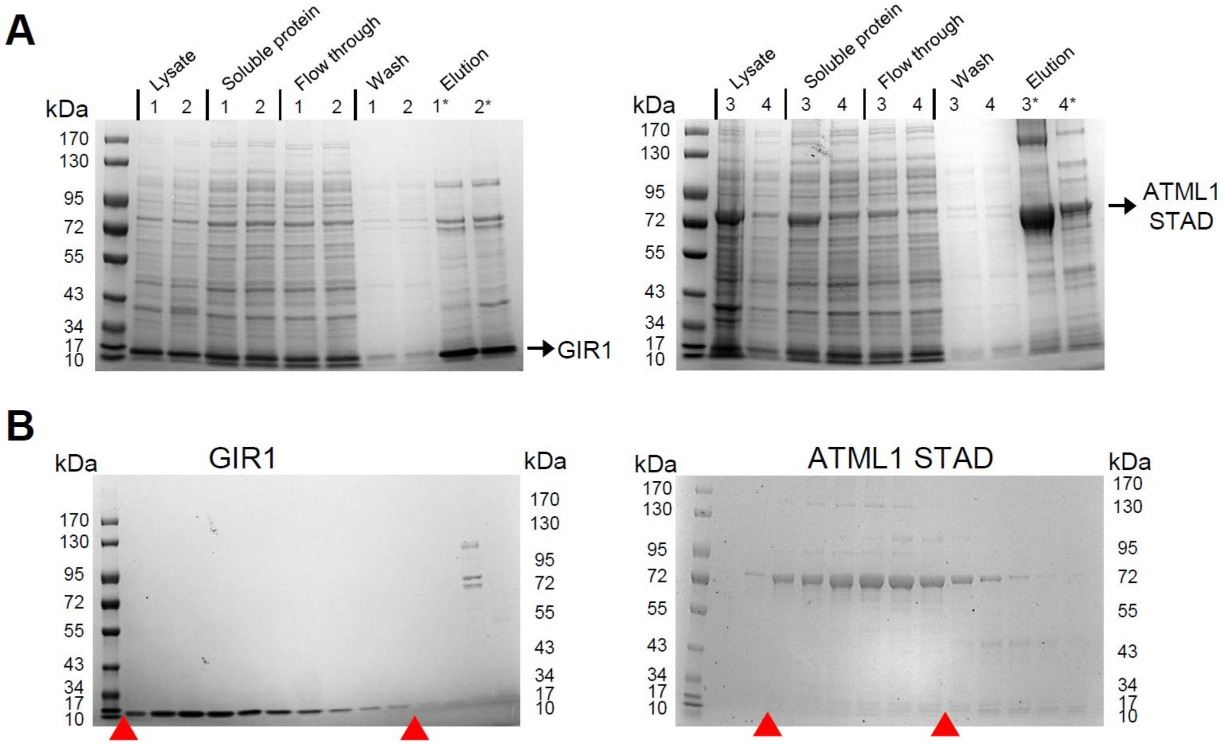

Fig. S4. Purification of recombinantly expressed GIR1 and ATML1 STAD wild-type and mutant proteins.

(A) Coomassie blue-stained gels on the left and right show protein purification of 6xHis-TEV-tagged **(1)** wild-type GIR1 and **(2)** mutant gir1^{C71A}, and 6xHis-MBP-TEV-tagged **(3)** wild-type ATML1 STAD and **(4)** mutant atml1 STAD^{D589L}, respectively. For each sample, the lysate, soluble supernatant, flow through from nickel affinity column, wash from column, and 500 mM imidazole elution are shown. Asterisks indicate samples taken for further purification by size exclusion chromatography. Arrows denote migration of the protein of interest. **(B)** SDS-PAGE of protein sample fractions purified by size exclusion chromatography. Fractions between red arrowheads indicate fractions that were pooled for final purified protein sample.

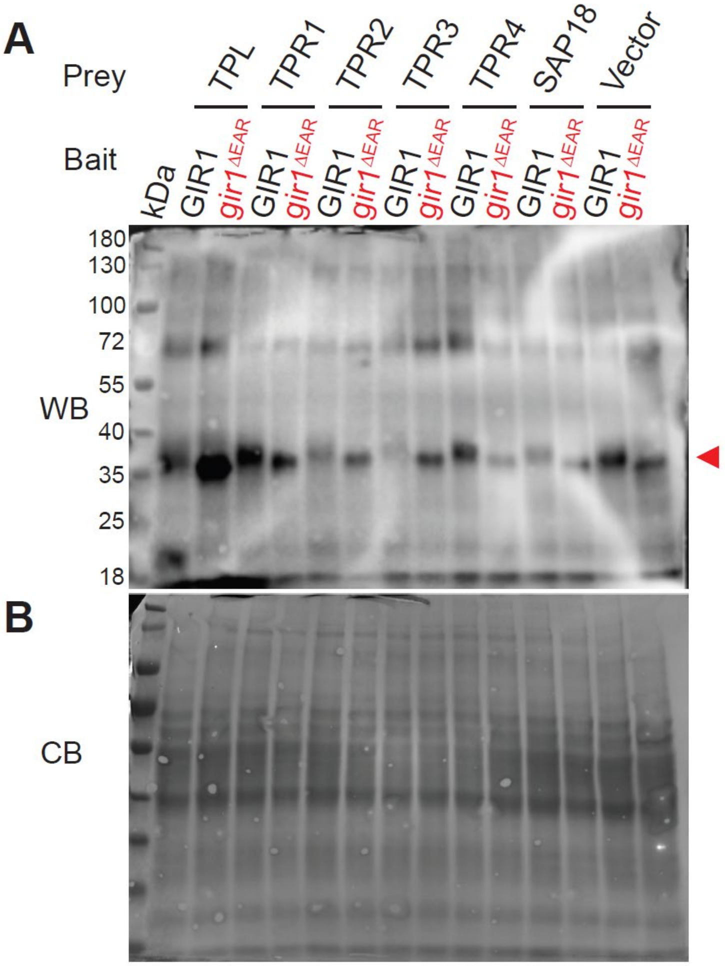

Fig. S5. Western blot of GIR1 proteins expressed in Y2H assays.

Western blot (WB, top) confirmed the Y2H bait protein expression for the assays shown in **Fig. 4C**. Wild-type GIR1 and mutant gir1$^{\Delta EAR}$ fused to the GAL4 DNA Binding Domain (DBD) were detected using an anti-GAL4 antibody. The red arrowhead indicates migration of GIR1 and gir1$^{\Delta EAR}$ GAL4 DBD fusion proteins. The grayscale image (bottom) shows total protein loading after Coomassie blue (CB) staining of the WB.

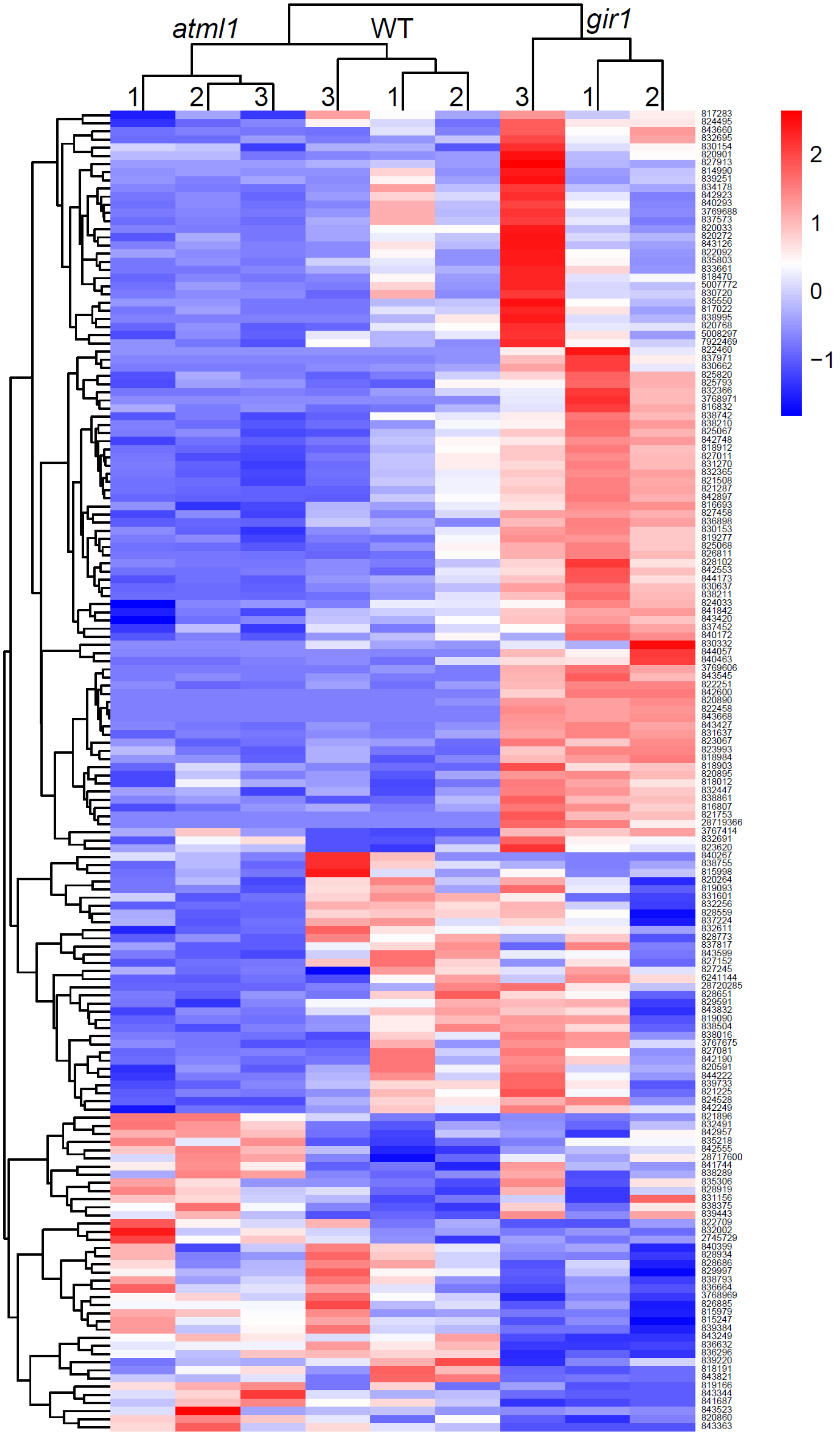

atml1
WT
gir1
1
2
3
3
1
2
3
1
2
2
1
0
−1

Fig. S6. Heatmap from RNA-seq data showing three biological replicates for each genotype.

Overview of DEGs in sepals from WT, *gir1*, and *atml1* plants. Gene expression levels are shown for n=3 biological replicates for each genotype, graded from low (blue) to high (red) ($P < 0.05$). A dendrogram depicting clustering of gene expression patterns is shown on the left of the heatmap. Gene IDs for the corresponding *Arabidopsis thaliana* genes are indicated on the right.

Aliphatic GSL biosynthesis - genes upregulated in *gir1*

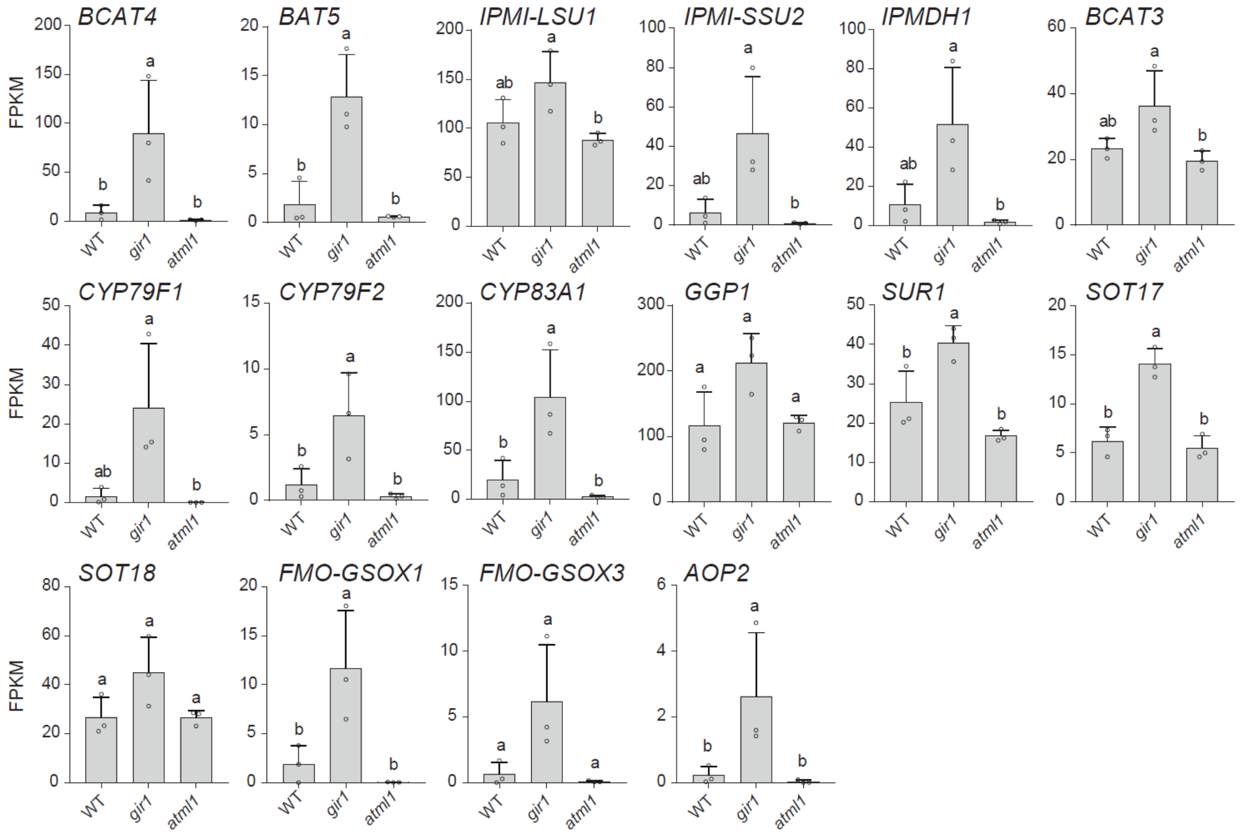

Aliphatic GSL biosynthesis - other

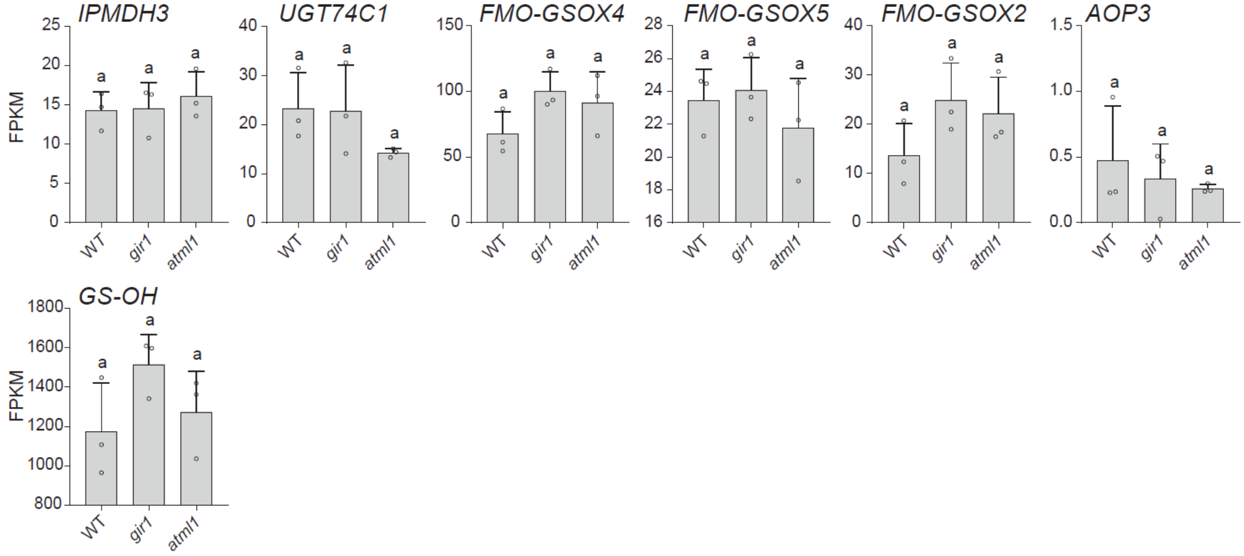

Fig. S7. RNA-seq data for additional aliphatic GSL biosynthesis genes. Related to Fig. 5.

The top group of graphs displays sepal RNA-seq data for aliphatic GSL biosynthesis genes upregulated in *gir1* mutant sepals, in comparison to wild type (WT) and *atml1* (*BCAT4, BAT5, IPMI-LSU1, IPMI-SSU2,*

IPMDH1, BCAT3, CYP79F1, CYP79F2, CYP83A1, GGP1, SUR1, SOT17, SOT18, FMOGSOX1, FMOGSOX3, AOP2). The bottom group of graphs shows sepal RNA-seq data for aliphatic GSL biosynthesis genes that are not regulated differentially in *gir1* mutants, in comparison to WT and *atml1*. (*IPMDH3, UGT74C1, FMO-GSOX4, FMOGSOX5, FMOGSOX2, AOP3, GS-OH*). FPKM, fragments per kilobase per million mapped reads. Genes are ordered within each group according to their approximate position in the GSL biosynthesis pathway, from left to right, top to bottom. Significant differences between genotypes are marked by letters (one-way ANOVA, Tukey's test, $p < 0.05$).

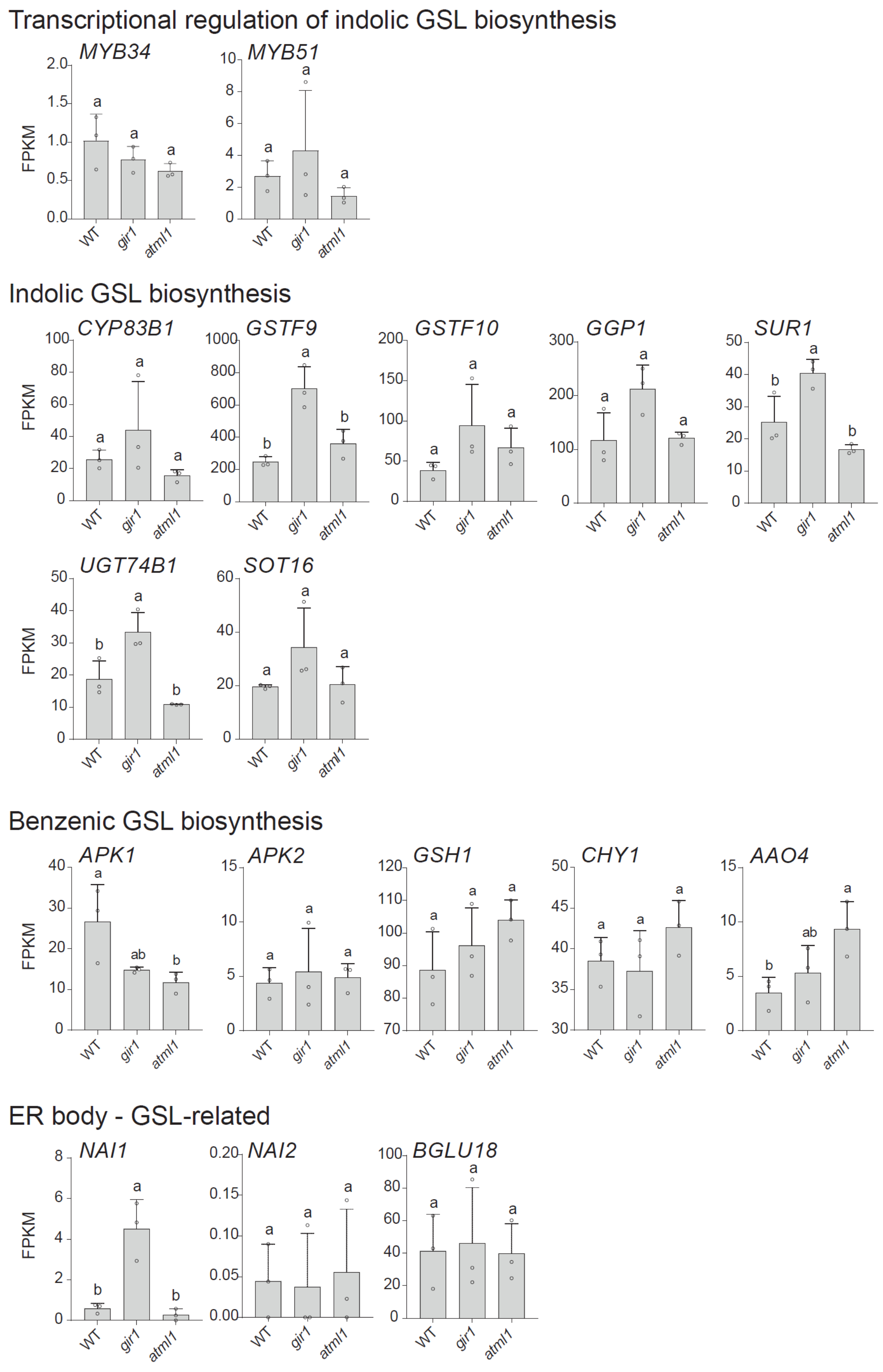

Fig. S8. Graphs of RNA-seq data for additional indolic GSL biosynthesis genes and other GSL pathway genes. Related to Fig. 5.

The top group of graphs displays sepal RNA-seq data for transcriptional regulators of the indolic GSL pathway (*MYB34, MYB51*). Transcripts of *MYB122* were not detected in 11/12 RNA-seq samples. The second group of graphs shows RNA-seq data for indolic GSL genes, all of which appear upregulated in *gir1* mutant sepals (*CYP83B1, GSTF9, GSTF10, GGP1, SUR1, UGT74B1, SOT16*). Note that *GGP1* and *SUR1* act in biosynthesis of both aliphatic and indolic GSLs. The third group of graphs show sepal RNA-seq data for benzenic GSL biosynthesis genes that are not upregulated in *gir1* mutants (*APK1, APK2, GSH1, CHY1, AAO4*). The bottom group of graphs show data for ER body and GSL-related genes (*NAI1, NAI2, BGLU18*). FPKM, fragments per kilobase per million mapped reads. Genes are ordered within each group according to their approximate position in the GSL biosynthesis pathway, from left to right, top to bottom. Error bars indicate SD. Significant differences between genotypes are marked by letters (one-way ANOVA, Tukey's test, $p < 0.05$).

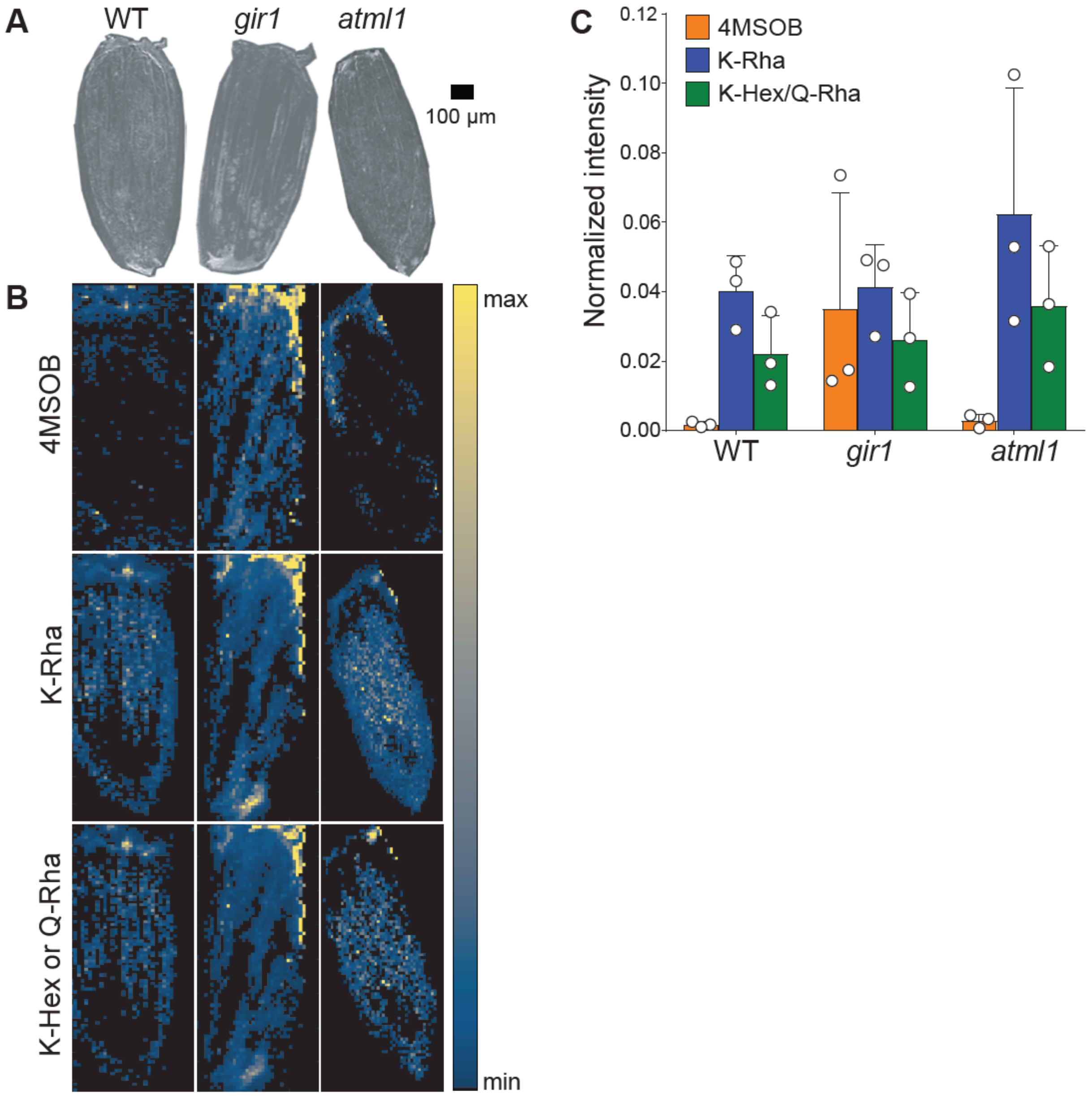

Fig. S9. MALDI-MS imaging of the sepal epidermis indicates high 4MSOB GSL levels in the *gir1* mutants.

(A) Optical images of *Arabidopsis thaliana* sepals from wild type (WT) in comparison to *gir1* and *atml1* mutants. **(B)** MALDI-MS ion images of the aliphatic GSL 4MSOB (top), and the flavonoids kaempferol rhamnoside (K-Rha) (middle), and kaempferol hexoside (K-Hex) or quercetin rhamnoside (Q-Rha) (bottom), shown to facilitate comparison with the GSL distribution. Ion intensities were normalized to the base peak (*m/z* 425.0129, $[9\text{-AA}+Au+Cl–H]^-$), and the maximum color scale was set to 1.0 across metabolites and genotypes. The color scale bar represents normalized ion intensity, and all ions are displayed as $[M–H]^-$. **(C)** Average normalized intensity of 4MSOB, K-Rha, and K-Hex or Q-Rha in each genotype at defined regions of interest (ROIs; n=3 biological replicates). Error bars indicate SD.

Table S1. Steady state constants and binding affinity of GIR1 to ATML1 STAD and atml1 STAD^{D589L}. Related to surface plasmon resonance (SPR) data presented in **Fig. 3G-H**. Equilibrium dissociation constants (K_D) are reported alongside the mean and SD for three injection series replicates (n=3). The data were fit to a steady-state model of binding. No fit or resulting K_D could be determined for the gir1^{C71A} mutant. Asterisk indicates that the K_D for the GIR1 over mutant STAD^{D589L} binding is significantly higher than the K_D for the GIR1 over wild-type STAD binding (*t*-test, two-tailed distribution, equal variance, p-value = 0.011).

**K_D (μM)**	**GIR1 over STAD**	**GIR1 over STAD^{D589L}**
Replicate 1	4.08	7.95
Replicate 2	4.49	8.24
Replicate 3	6.04	9.93
Mean	4.87	8.71 *
SD	1.03	1.07

Table S2. **Summary of expression of GSL biosynthesis genes in *gir1* sepals.** The fold-change (FC) difference of the average expression between the three replicates of GSL biosynthesis genes [3] in *gir1* was compared to wild type. Genes are ordered based on level of FC upregulation. Related to **Fig. 5**.

**Expression Differences of Glucosinolate Biosynthesis Pathway Genes between *gir1* vs. WT**			
Gene	Gene ID	Locus	*gir1* vs. WT FC
**Aliphatic Pathway Genes**			
MYB29	830662	At5g07690	16.0
CYP79F1	838211	At1g16410	14.9
AOP2	828102	At4g03060	12.0
MAM1	832365	At5g23010	10.9
BCAT4	821508	At3g19710	10.4
FMO-GSOX3	842553	At1g62560	9.5
MAM3	832366	At5g23020	7.6
IPMI SSU2	818912	At2g43100	7.3
BAT5	826811	At4g12030	6.9
FMO-GSOX1	842897	At1g65860	6.2
CYP79F2	838210	At1g16400	5.4
CYP83A1	827011	At4g13770	5.2
IPMI SSU3	825068	At3g58990	5.1
IPMDH1	831270	At5g14200	4.8
GSTU20	844173	At1g78370	4.5
SOT17	838440	At1g18590	2.3
GGP1	829176	At4g30530	1.8
FMO-GSOX2	842551	At1g62540	1.8
SOT18	843749	At1g74090	1.7
BCAT3	824130	At3g49680	1.6
SUR1	816585	At2g20610	1.6
MYB28	836263	At5g61420	1.6
FMO-GSOX4	842554	At1g62570	1.5
IPMI LSU1	826975	At4g13430	1.4
GS-OH	817083	At2g25450	1.3
IPMDH3	840006	At1g31180	1.0
UGT74C1	817736	At2g31790	1.0
FMO-GSOX5	837766	At1g12140	1.0
AOP3	828104	At4g03050	0.7
**Indolic and Benzenic Pathway Genes**			
CYP79B2	830154	At4g39950	7.2
CYP79B3	816765	At2g22330	3.8
GSTF9	817636	At2g30860	2.8
GSTF10	817637	At2g30870	2.4
GGP1	829176	At4g30530	1.8
UGT74B1	839022	At1g24100	1.8
SOT16	843750	At1g74100	1.8
CYP83B1	827011	At4g13770	1.7
SUR1	816585	At2g20610	1.6
**Co-substrate Pathway Genes**			
AAO4	839488	At1g04580	1.5
APK2	830153	At4g39940	1.2
GSH1	828409	At4g23100	1.1
CHY1	836724	At5g65940	1.0
APK1	815963	At2g14750	0.6

Table S3. Names and chemical properties of glucosinolates described in this study. The glucosinolates (GSLs) are classified as aliphatic (top) or indolic (bottom).

Abbreviation	Full Name	Common Name	Chemical Formula	Exact Mass (Da)	Molecular Weight (g/mol)
**Aliphatic GSL**					
3MTP	3-Methylthiopropyl	Glucoiberverin	C11H21NO9S3	407.03784477	407.5
4MTB	4-Methylthiobutyl	Glucoerucin	C12H23NO9S3	421.05349483	421.5
5MTP	5-Methylthiopentyl	Glucoberteroin	C13H25NO9S3	435.0691	435.5
6MTH	6-Methylthiohexyl	Glucolesquerellin	C14H27NO9S3	449.0848	449.6
7MTH	7-Methylthioheptyl		C15H29NO9S3	463.10044502	463.6
8MTO	8-Methylthiooctyl		C16H31NO9S3	477.11609509	477.6
3MSOP	3-Methylsulfinylpropyl	Glucoiberin	C11H21NO10S3	423.0328	423.5
4MSOB	4-Methylsulfinylbutyl	Glucoraphanin	C12H23NO10S3	437.0484	437.5
5MSOP	5-methylsulfinylpentyl	Glucoalyssin	C13H25NO10S3	451.0641	451.5
6MSOH	6-Methylsulfinylhexyl	Glucohesperin	C14H27NO10S3	465.07970958	465.6
7MSOH	7-Methylsulfinylheptyl		C15H28NO10S3	478.08753461	478.6
8MSOO	8-Methylsulfinyloctyl	Glucohirsutin	C16H31NO10S3	493.11100971	493.6
3OHP	3-Hydroxypropyl		C10H19NO10S2	377.04503815	377.4
4OHB	4-hydroxybutyl		C11H21NO10S2	391.06068821	391.4
Benzyl	Benzyl	Glucotropaeolin	C14H19NO9S2	409.05012353	409.4
3BZOP	3-Benzoyloxypropyl	Glucomalcomiin	C17H23NO11S2	481.07125290	481.5
4BZOB	4-Benzoyloxybutyl		C18H25NO10S2	479.09198834	479.5
**Indolic GSL**					
I3M	Indol-3-ylmethyl	Glucobrassicin	C16H20N2O9S2	448.06102257	448.5
1OHI3M	1-hydroxy-indol-3-ylmethyl		C16H20N2O10S2	464.0559	464.5
4OHI3M	4-hydroxy-indol-3-ylmethyl		C16H19N2O10S2	463.04811215	463.5
1MOI3M	1-methoxy-indol-3-ylmethyl		C17H22N2O10S2	478.07158725	478.5
4MOI3M	4-methoxy-indol-3-ylmethyl		C17H22N2O10S2	478.07158725	478.5

Table S4. Oligonucleotides used in this study.

Primers for genotyping mutants.	
gir1_SALK_089888_F	CTTTTTCTGGGGATCAGGATC
gir1_SALK_089888_R	AAGGCCTAAGCTCAAGCAATC
gir2_SALK_048394_F	CAACAAAATGGAATAACAGTTAAAGC
gir2_SALK_048394_R	CAGGTCTTCTTCTTGCGTCTG
atml1-3_F	CAGGCAGAAGAAAATCGAGAT
atml1-3_R	GAAACCAGTGTGGCTATTGTT
LbB1.3 (T-DNA for *gir1, 2*)	ATTTTGCCGATTTCGGAAC
LBb1 (T-DNA for *atml1-3*)	GCGTGGACCGCTTGCTGCAACT
Primers for TOPO directional cloning. Additional CACC sites in forward primers required for directional cloning are bolded while stop codons are in blue. Alternative bases used for mutant generation are red.	
Topo_ATML1_F	**CACC** ATG TAT CAT CCA AAC ATG TTC G
ATML1_C_R_new	AT CGA TTA GGC TCC GTC GCA G
ATML1_START_F	**CACC** ATA CCT TCT GAG GCT GAT AAG
ATML1_START_R	CTA CAT GGA ACT GGC GAG CCG
ATML1_STAD_F	**CACC** GCC AGC AAC ATT CCG G
GIR1_cDNA_F	**CACC** ATG AGT CGA AGA AGT CC
GIR1_cDNA_R	TCA TCA GTT CCT TCG AGT C
TPL_F	**CACC** ATG TCT TCT CTT AGT AG
TPL_R	TCA TCT CTG AGG CTG ATC AG
Topo_TPR1_F	**CACC** ATG TCT TCT CTG AGC AG
Topo_TPR1_R	TCA TCT CTG AGG CTG GTC AG
atml1_K547E_F	CCG GGA AGA CCT CCA GGC ATC G
atml1_K547E_R	ATC ATC CAT GCT CTC TCG GGT CAT GAC C
atml1_D589L_F	CTT ATA CTT TCC AAT GGA GGC
atml1_D589L_R	CCA CTC GCT TCT TGA GTT TTC ATC
atml1_N593A_F	GCT GGA GGC TTG GTT CAA GAA ATG
atml1_N593A_R	GGA AAG TAT ATC CCA CTC GCT TC
atml1_N627A_F	GCC ATG TTG ATC TTA CAA GAA AGT TG
atml1_N627A_R	GCT CTG CCC TGA GTT CCC
atml1_E633L_F	TTA AGT TGT ACG GAC GCA TCA GGG
atml1_E633L_R	TTG TAA GAT CAA CAT GTT GCT CTG CCC
atml1_K733E_F	GAA CTC TCT CTC GGT TCA GTT GC
atml1_K733E_R	AGC GGT AGG AAC AGA GTC AAC AAG
gir1_R23L;R30L_F	TTG ATG GTT CGA TCT CCA AGC CTC TCA GCG
gir1_R23L_R	CCT CTG GCT TGA CGT AGG CGG CG
gir1_R30E_F	GAG TCA GCG ACG ACT TCA CCG ACC
gir1_R30L_R	GCT TGG AGA TCG AAC CAT CCT CCT CTG G
Primers for DNA assembly to clone into SR54 vector. Sequence overlapping with SR54 colored in blue while restriction sites are indicated in red. Bases used for alanine mutations are bolded.	
SR54_gir1_complement_F	AACGACGGCCAGTGCC AAGCTT GATGAAATGCATGAACGTACG
SR54_gir1_complement_R	CACCGCGGTGGAGCTC GGTACC GTTTTTAGGCCTGTGATTAGC
gir1_C87A_F	**GC**C CCT AAA TGC AAA AGC ACC G
gir1_C87A_R_Ass	GT GCT TTT GCA TTT AGG G**GC** TTT AGG GTC GTC
gir1_C71A_F	**GC**T CCT CGT TGT CTC ATG TAC GTT ATG C
gir1_C71A_R_Ass	TACATGAGACAACGAGG A**GC** TCC GAC AAG AAC C
gir1_Del_EAR_F	AATCTCTCGCCGCCTACG
gir1_Del_EAR_R	CTT TGG ACT TCT TCG ACT CAT
gir1_Del_21_18_EAR_F	ATGAGTCGAAGAAGTCCAAA GAA TCT CTC GCC GCC TAC G
GIR1_TPL_R	C TGG ATT TGG CCT TGG GTT CCT TCG AGT CTT GCG AC
TPL_C_Term_F	CCA AGG CCA AAT CCA GAT ATC
TPL_R	TCA TCT CTG AGG CTG ATC AG
TPL_C_GIR1_3UTR_F	CAGCCTCAGAGATGATGAAGGACCAGTAAAATCAAAATCCTG

Primers for cloning cDNAs into plasmids for protein production. (Restriction sites, red; cDNA sequences, blue)	
pET-His6-MBP-TEV-Lic_SspI_ATML1_STAD_F	ACCTGTACTTCCAATCC AATATT AGTCCTGAGGGGAGAAA
pET-His6-MBP-TEV-Lic_SspI_ATML1_STAD_R	ATCCGTTATCCACTTCC AATATT TTAGGCTCCGTCGCA
pT7HMT_BamHI_GIR1_F	TACTTCCAGGGGTCGACA GGATCC ATGAGTCGAAGAAGTCC
pT7HMT_NotI_GIR1_R	GTGGTGGTGCTCGAGT GCGGCCGC TCAGTTCCTTCGAGTCTTG

SI Dataset Legends

Dataset S1 (separate sheet in xls file). Comprehensive RNA-seq data from Arabidopsis sepals. Raw read counts, followed by fpkm (fragments per kilobase of transcript for million mapped reads) normalized read counts are show for each of the three replicates for wild type (WT), *gir1* and *atml1* sepal RNA. Log2 fold-change values, p values, and adjusted p values (padj) are indicated for each binary genotype comparison. The following gene metrics for each gene ID are given: gene name, chromosomal location (chr), start position, end position, strand (+ or -), length, biotype, gene description, gene family.

Dataset S2 (separate sheet in xls file). List of DEGs that are up-regulated in *gir1* compared to wild-type sepals (padj<0.05, log2FC>1).

Dataset S3 (separate sheet in xls file). List of DEGs that are down-regulated in *gir1* compared to wild-type sepals (padj<0.05, log2FC>1).

Dataset S4 (separate sheet in xls file). List of DEGs that are up-regulated in *atml1* compared to wild-type sepals (padj<0.05, log2FC>1).

Dataset S5 (separate sheet in xls file). List of DEGs that are down-regulated in *atml1* compared to wild-type sepals (padj<0.05, log2FC>1).

Dataset S6 (separate sheet in xls file). List of DEGs that are up-regulated in *gir1* compared to *atml1* sepals (padj<0.05, log2FC>1).

Dataset S7 (separate sheet in xls file). List of DEGs that are down-regulated in *gir1* compared to *atml1* sepals (padj<0.05, log2FC>1).

Dataset S8 (separate sheet in xls file). Chemical analysis of GSL contents of mutants performed by HPLC-DAD. Quantification of GSL is shown for extracts from gir*1*, *gir2*, and *atml1* mutant sepals and buds without (w/o) sepals in comparison to wild type (WT). Full names of GSL compounds and information on their properties are given in **Table S3**. GSLs were quantified from absorbance peaks.

Dataset S9 (separate sheet in xls file). Chemical analysis of GSL contents of transgenic lines performed by HPLC-DAD. Quantification of GSL is shown for extracts from transgenic *gir1* mutant lines carrying stable wild-type *pGIR1:GIR1* and mutant *pGIR:gir1*^{C71A} transgenes. Extracts from sepals versus buds without (w/o) sepals are listed. Full names of GSL compounds and information on their properties are given in **Table S3**. GSLs were quantified from absorbance peaks.

SI References

1. Robichaud, G., Garrard, K.P., Barry, J.A., and Muddiman, D.C. (2013). MSiReader: an open-source interface to view and analyze high resolving power MS imaging files on Matlab platform. J Am Soc Mass Spectrom *24*, 718-721. 10.1007/s13361-013-0607-z.

2. Palmer, A., Phapale, P., Chernyavsky, I., Lavigne, R., Fay, D., Tarasov, A., Kovalev, V., Fuchser, J., Nikolenko, S., Pineau, C., Becker, M., and Alexandrov, T. (2017). FDR-controlled metabolite annotation for high-resolution imaging mass spectrometry. Nat Methods *14*, 57-60. 10.1038/nmeth.4072.

3. Sonderby, I.E., Geu-Flores, F., and Halkier, B.A. (2010). Biosynthesis of glucosinolates--gene discovery and beyond. Trends Plant Sci *15*, 283-290. 10.1016/j.tplants.2010.02.005.
